# Integrative Analysis and Validation of a Cancer-associated Fibroblasts Senescence-related Signature for Risk Stratification and Therapeutic Prediction in Esophageal Squamous Cell Carcinoma

**DOI:** 10.7150/jca.100430

**Published:** 2024-09-09

**Authors:** Han Zhang, Kunqiao Hong, Qi Song, Beibei Zhu, Gang Wu, Baoping Yu

**Affiliations:** 1 Department of Gastroenterology, Renmin Hospital of Wuhan University, Wuhan, Hubei, PR China.; 2 Key Laboratory of Hubei Province for Digestive System Diseases, Wuhan, Hubei, PR China.; 3 Department of Cardiology, Renmin Hospital of Wuhan University, Wuhan, Hubei, PR China.

**Keywords:** Cancer-associated fibroblasts, Cellular senescence, Esophageal squamous cell carcinoma, Risk signature, Single-cell RNA sequencing

## Abstract

Cellular senescence is closely associated with cancer development and progression. There is ample evidence that tumor stromal cells, especially cancer-associated fibroblasts (CAFs) undergo senescence in response to various stimuli. However, the possible biological roles and prognostic significance of senescent CAFs in esophageal squamous cell carcinoma (ESCC) remain unexplored. In this study, we found that CAFs exhibited a significantly higher level of cellular senescence than other cell clusters at the single-cell level. Then, we constructed a CAFs senescence-associated risk model with 7 genes (*GEM*, *SLC2A6*, *CXCL14*, *STX11*, *EFHD2*, *PTX3*, and *HCK*) through Cox regression and LASSO analysis. Kaplan-Meier survival analysis revealed that the risk model was significantly correlated with worse prognosis in training and validation cohorts. Subsequent analysis indicated that the risk model was an independent prognostic factor. In addition, the signature showed a distinct negative correlation with immune cell infiltration and immunotherapy responses. *In vitro* experiments showed remarkably higher mRNA and protein levels of prognosis-related genes (*STX11* and *EFHD2*) in senescent CAFs than control group, consistent with the bioinformatics analysis results. Moreover, senescent CAFs significantly promoted ESCC cell proliferation and migration as shown by CCK-8 and scratch assays. In conclusion, our study identified a novel CAFs senescence-based classifier that may help predict prognosis of ESCC, and a thorough characterization of the signature could also be helpful in evaluating the response of ESCC to anti-tumor therapies and provide meaningful clinical options for cancer treatment.

## Introduction

Esophageal carcinoma (EC) is one of the most common digestive system cancers, ranking eighth in incidence and sixth in mortality among all cancer types worldwide 1. EC is mainly categorized into two subtypes: esophageal squamous cell carcinoma (ESCC) and esophageal adenocarcinoma 2. ESCC is the major subtype in Eastern Asia, particularly in China, comprising about 90% of EC cases 3, 4. Given the lack of clinical signs in the initial phase, ESCC patients are usually diagnosed at an advanced stage, leading to poor life quality and adverse survival outcomes 5. Even with advances in treatments like surgery, radiotherapy, chemotherapy, and immunotherapy, less than 20% of ESCC patients survive longer than five years 6. Consequently, searching and developing new molecular indicators are crucial for precisely forecasting the clinical behavior and prognosis for ESCC.

Cellular senescence refers to a stable cell-cycle arrest, which can be induced by a range of insults, containing telomere shortening, oncogenic activation, oxidative stress, and DNA damage 7, 8. Senescent cells experience numerous phenotype shifts, such as metabolic reprogramming, morphological alterations, and chromatin reorganization, and acquire a proinflammatory phenotype termed the senescence-associated secretory phenotype (SASP), characterized by the secretion of growth factors, matrix metalloproteinases, cytokines, and chemokines 9, 10. Recent studies demonstrate that senescent cells exacerbate tumor growth fueled by the senescent microenvironment 11-13. However, the distribution of cellular senescence within tumors and adjacent stromal areas remains unclear. With the continuous progress of sequencing technology, we now could characterize cellular senescence among different cell populations from the single-cell level.

The tumor microenvironment (TME), where tumor cells grow and evolve, has an inseparable relationship with tumorigenesis 14, 15. TME is composed of multiple cells, including but not limited to immune cells, endothelial cells, cancer-associated fibroblasts (CAFs), pericytes, and various extracellular matrix components 16, 17. It has been reported that cellular senescence in stromal cells, especially in CAFs, has a profound effect on initiation and progression of tumor. For example, senescent CAFs release SASP factors to facilitate pancreatic cancer cell proliferation 18. Cellular senescence in CAFs promotes breast cancer progression through establishing an immunosuppressive environment 19. However, the characteristics of senescent CAFs and their effect on treatment response and prognosis of ESCC are still unexplored.

In the present study, we acquired single-cell RNA sequencing (scRNA-seq) data from the GEO dataset to evaluate the senescence status of each cell subcluster. We found that CAFs exhibited the highest cellular senescence level and established a CAFs senescence-related signature for ESCC. We further analyzed the clinical significance and immune landscape underlying the signature. Finally, the bioinformatics analysis results were validated by *in vitro* experiments. Our findings shed insights into the senescence-related biomarker discoveries and provided a new perspective for predicting prognosis in ESCC.

## Materials and methods

### Data collection

All sequencing data used in the study was downloaded from public databases. scRNA-seq data was downloaded from GEO datasets (GSE160269). RNA sequencing (RNA-seq) data used for training of risk signature was downloaded from GEO datasets (GSE53624) and the validation dataset were downloaded from TCGA. The scRNA-seq dataset contains 60 tumor samples and 4 normal samples. GSE53624 has 119 tumor samples and 119 normal samples, among which we chose tumor samples as research objects. There are a total of 94 samples in the TCGA cohort, all of which are involved for validation.

### scRNA-seq data analysis

The UMI matrix and cell information were downloaded separately and integrated into an AnnData object using Scanpy (v1.9.1) package. The re-analysis pipeline of scRNA-seq was built based on the Scanpy package to save the resource consumption 20. In brief, single cells with less than 300 genes or more than 6000 genes were discarded. The percentage of rRNA and mitochondrial genes were calculated, and single cells with more than 10% mitochondrial genes were filtered. Finally, about 200,000 cells were obtained for further exploration. We applied 3000 highly variable genes to define cell clusters by PCA and leiden functions provided by Scanpy. Sc.tl.umap function was adopted for dimension reduction and visualization. The immune and non-immune cell clusters were separated by marker gene PTPRC (CD45), and cell types were annotated based on the canonical markers.

### Senescent CAF-related genes identification

CAFs were isolated and re-clustered. To avoid the bias caused by different samples, the batch effect was removed using harmony method 21. In detail, we extracted the expression matrix of all CAFs from the raw matrix, removed the batch effect using harmony and did the standard scanpy pipeline afterwards. Then, CAFs were re-clustered by sc.tl.leiden function and labeled with 6 markers, including *ACTA2*, *NOTCH3*, *S100A4*, *FAP*, *TGF-β1*, and *PDGFRB* 22-24. The senescent status of cells was assessed using sc.tl.score_genes function and the established senescent signature FRIDMAN.SENESCENCE.SIGNATURE obtained from MsigDB was used as reference 25, 26. Cells with a score higher than the median were considered as senescent cells. The rank_genes_groups of Scanpy was adopted to determine genes that were differently expressed between senescent and normal cells.

### Function annotation and enrichment analysis

The function annotation and enrichment analysis of interested genes were completed using ClusterProfiler (v4.10.1) 27. The gene function annotation analyses were based on Gene Ontology (GO), while the pathway enrichment analyses were using Kyoto Encyclopedia of Genes and Genomes (KEGG) as the background database. Terms with qvalue < 0.1 were recognized as significantly enriched ones. Specific pathways were analyzed using the Gene Set Enrichment Analysis (GSEA) software (v4.3.3) for calculating enrichment scores 28, 29, and the pathway-related information was obtained from MSigDB.

### Cell-cell communication analysis

The CellChat (v1.5.0) package was applied to quantitatively detect the cell-cell crosstalk in the tumor microenvironment 30, which provides well-formed interaction network using the feature of ligands, receptors and relevant cofactors. The officially recommended parameters were adopted to calculate the number and strength of interactions between all cell types, while the visualization functions of CellChat were optimized to show the detailed ligand-receptors according to signaling pathways.

### Construction and validation of CAFs senescence-related signature

We conducted a univariate Cox regression analysis on the hub genes in GSE53624 cohort using R package “survival” (v3.5.8). A total of 17 genes with *p*-value < 0.2 were identified as having a notable correlation with the overall survival (OS) of ESCC patients. Then, these genes were involved in the least absolute shrinkage and selection operator (LASSO) analysis, after which 7 genes were recognized as key prognostic genes. Subsequently, the multivariate Cox regression model, focusing on major prognostic genes, was developed utilizing the R package “glmnet” (v4.1.8) 31. The relationship between genes and OS was visualized by R package “forestplot” (v3.1.3). Finally, a risk model was developed by simultaneously considering the gene expression conditions and relevant regression coefficients of each sample. The risk score model can be described as:



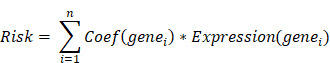



Patients were split into two groups based on the median risk score of all samples, and those with higher score were classified as high-risk group. Using R package “survminer” (v0.4.9), a Kaplan-Meier curve was developed to demonstrate the variance in survival rates between two risk groups. Receiver operating characteristic (ROC) and the corresponding area under the ROC curve (AUC) were summarized to depict the sensitivity and specificity of the risk model.

### Construction of a prognostic nomogram based on the risk model

Using both univariate and multivariate Cox regression, a novel nomogram was constructed. The performance of the nomogram and clinical variables were evaluated using the calibration curve and decision curve analysis (DCA). The calibration and ROC curves were generated using R packages “rms” (v6.8.0) and “timeROC” (v0.4.0), respectively 32.

### Stromal and immune cell infiltration analysis

The immune cell infiltration profiling was conducted using CIBERSORT 33. The R package “estimate” (v4.0.0) was adopted to calculate indexes including two immune infiltration scores (stromal score and immune score) and tumor purity.

### Evaluation of correlation between signature and the efficacy of immunotherapy

The application of the signature in immunotherapy was measured in two cohorts underwent immune checkpoint inhibitor therapy. IMvigor210 is a cohort of patients with advanced urothelial carcinoma underwent anti-PD-L1 immunotherapy. GSE78220 cohort contains patients with melanoma received anti-PD-1 immunotherapy.

### Drug sensitivity prediction

To extend the clinical application of the novel signature, oncoPredict (v0.2.0) program was performed using CTRPv2 as reference to evaluate the drug sensitivity of patients from two subgroups. The drugs showing a significantly low half maximal inhibitory concentration (IC50) in patients with high-risk scores were regarded as sensitive, whereas those with high IC50 were regarded as resistant.

### Cell culture

The fetal lung fibroblasts (MRC-5) were acquired from Procell Life Science & Technology Co, Ltd (Wuhan, China). ESCC cell lines (KYSE-150 and ECA-109) were acquired from Shanghai Cell Bank (Chinese Academy of Sciences, Shanghai, China). All cells were cultured in RPMI-1640 (Gibco, Gaithersburg, MD, USA), supplemented with 10% fetal bovine serum (Gibco, Gaithersburg, MD, USA) and 1% penicillin/streptomycin (Beyotime, Shanghai, China). Cells were routinely grown in a humidified atmosphere with 5% CO_2_ at 37 °C.

### Induction of senescence and preparation of conditioned media (CM)

MRC-5 cells were treated with 500 μM H_2_O_2_ for 2 h. Then, the cells were washed and incubated with complete culture media for additional 7 days. To collect CM, MRC-5 cells were cultured with serum-free media for 48 h. Then, CM was collected, centrifuged, and stored at -80 °C.

### Senescence associated β-galactosidase (SA-β-gal) staining

The senescence condition of cells was assessed using the SA-β-gal Staining Kit (Beyotime, Shanghai, China). The post-treated cells were washed, fixed with 4% paraformaldehyde, and stained with the staining working solution at 37 °C overnight in the absence of CO_2_. The cells were checked with a microscope (Olympus IX71, Tokyo, Japan). Total and positive cells were calculated from five random fields using the ImageJ software.

### RNA extraction and quantitative real-time polymerase chain reaction (qRT-PCR)

RNA was isolated using TRIzol reagent (Invitrogen, CA, USA). Complementary DNA was transcribed using a PrimeScript RT Master Kit (Takara, Ohtsu, Japan). SYBR Green (Takara, Ohtsu, Japan) was used to conduct qRT-PCR on a Bio-Rad CFX PCR machine. mRNA levels were normalized to the relative quantity of GAPDH and were calculated according to the 2^-ΔΔCt^ method. The primers used for qRT-PCR were listed as following:

*STX11*: 5′-GTAAGTGGGACGTGTTTTCCG-3′(forward),

5′-CTCGATGACGTTCAGGGTGT-3′ (reverse);

*EFHD2*: 5′-CCCCTACACCGAGTTCAAGG-3′(forward),

5′-TGGACTGCAGCTCCTTGAAG-3′ (reverse);

*GAPDH*: 5′-GGAGCGAGATCCCTCCAAAAT-3′(forward),

5′-GGCTGTTGTCATACTTCTCATGG-3′ (reverse).

### Western blot

Cells were lysed with RIPA (Beyotime, Shanghai, China) containing 1 mM PMSF (Beyotime, Shanghai, China) on ice. Then, the cell lysates were sonicated and centrifuged at 4 ℃, 12000 rpm, 15 min to collect supernatants. After denaturation at 100 ℃ for 10 min, protein was subjected to 10% SDS-PAGE and transferred to PVDF membrane (Millipore, MA, USA). The membranes were blocked in milk at room temperature for 2 h, and incubated with the following primary antibodies at 4 °C overnight: anti-GAPDH (#60004-1-Ig, Proteintech, Wuhan, China), anti-STX11 (#13301-1-AP, Proteintech, Wuhan, China), and anti-EFHD2 (#83264-5-RR, Proteintech, Wuhan, China). After incubation, the membranes were washed with TBST and then incubated with the following secondary antibodies at room temperature for 1 h: HRP-conjugated anti-Mouse IgG (#SA00001-1, Proteintech, Wuhan, China) and HRP-conjugated anti-Rabbit IgG (#SA00001-2, Proteintech, Wuhan, China). Subsequently, the membranes were washed with TBST and visualized using ECL luminescent solution (Epizyme, Shanghai, China) on an imaging system (Bio-rad, CA, USA).

### Cell counting kit-8 (CCK-8)

ESCC cells were implanted into a 96-well plate, and the culture medium was replaced by different CM after overnight culture. At each time point, the medium was replaced with CCK-8 reagent (Biosharp, Hefei, China). After incubation at 37 °C for 1 h, the absorbance was detected using a microplate reader (Thermo, Waltham, USA) at 450 nm.

### Wound healing assay

ESCC cells were plated in a 6-well plate and cultured overnight to reach about 80% confluence. Using a sterile 200 μL pipette tip, a linear wound was created, and the cell debris was gently washed. After that, the cells were subjected to different treatments, and a microscope (Olympus IX71, Tokyo, Japan) was used to take images at 0 and 24 hours after scratching. The width of scratches was quantified using the ImageJ software.

### Statistical analysis

All statistical analyses were performed using R software (v4.3.0). The Wilcoxon test was employed for comparing two groups. The correlation between two factors was depicted using Pearson correlation algorithm. We assessed survival variations through Kaplan-Meier curves and utilized the Log-rank test to determine statistical significance, using *p*-value < 0.05 as the cutoff.

## Results

### The single-cell profiling of ESCC

**Figure 1:**
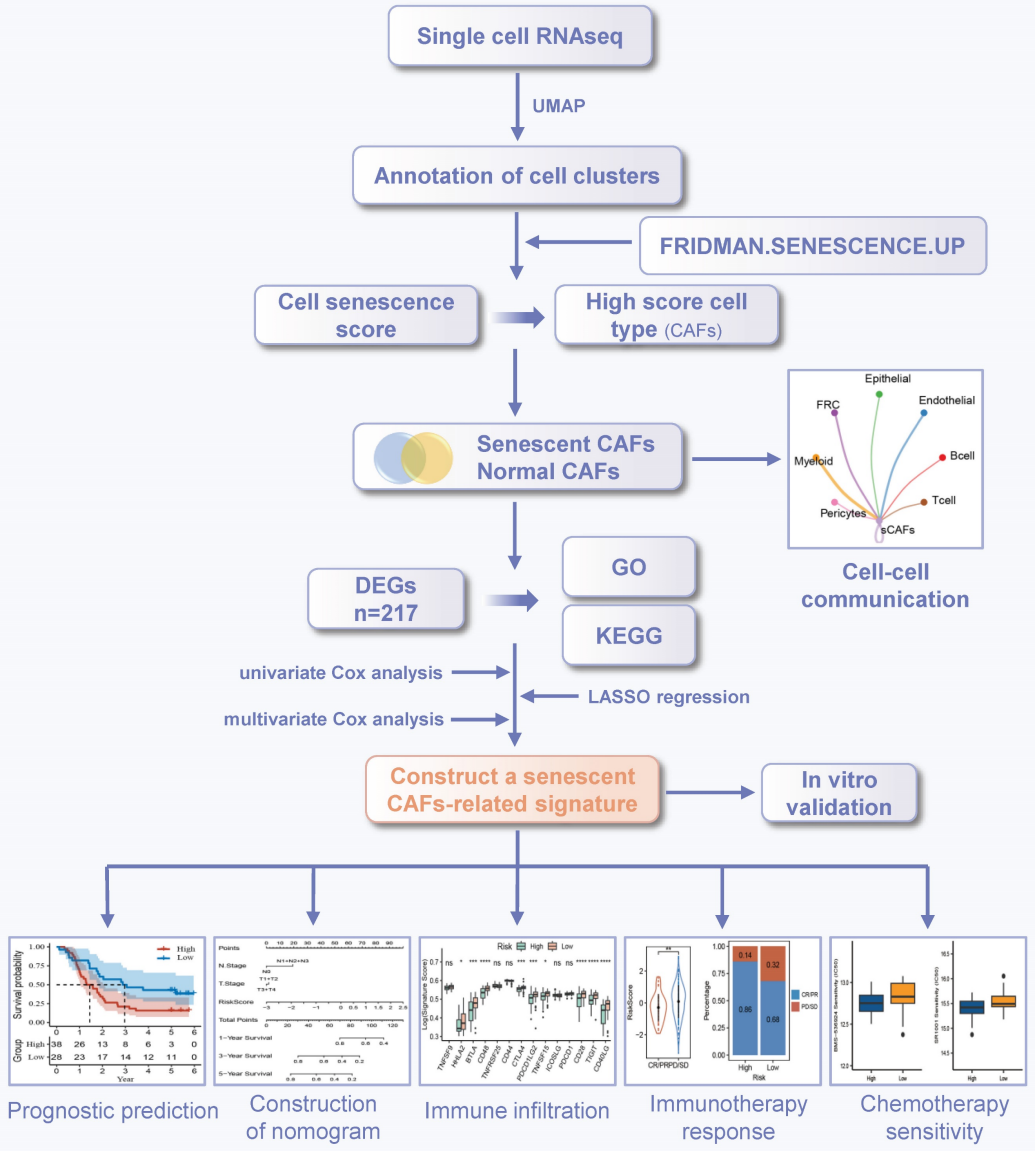
The workflow of this study.

**Figure 2:**
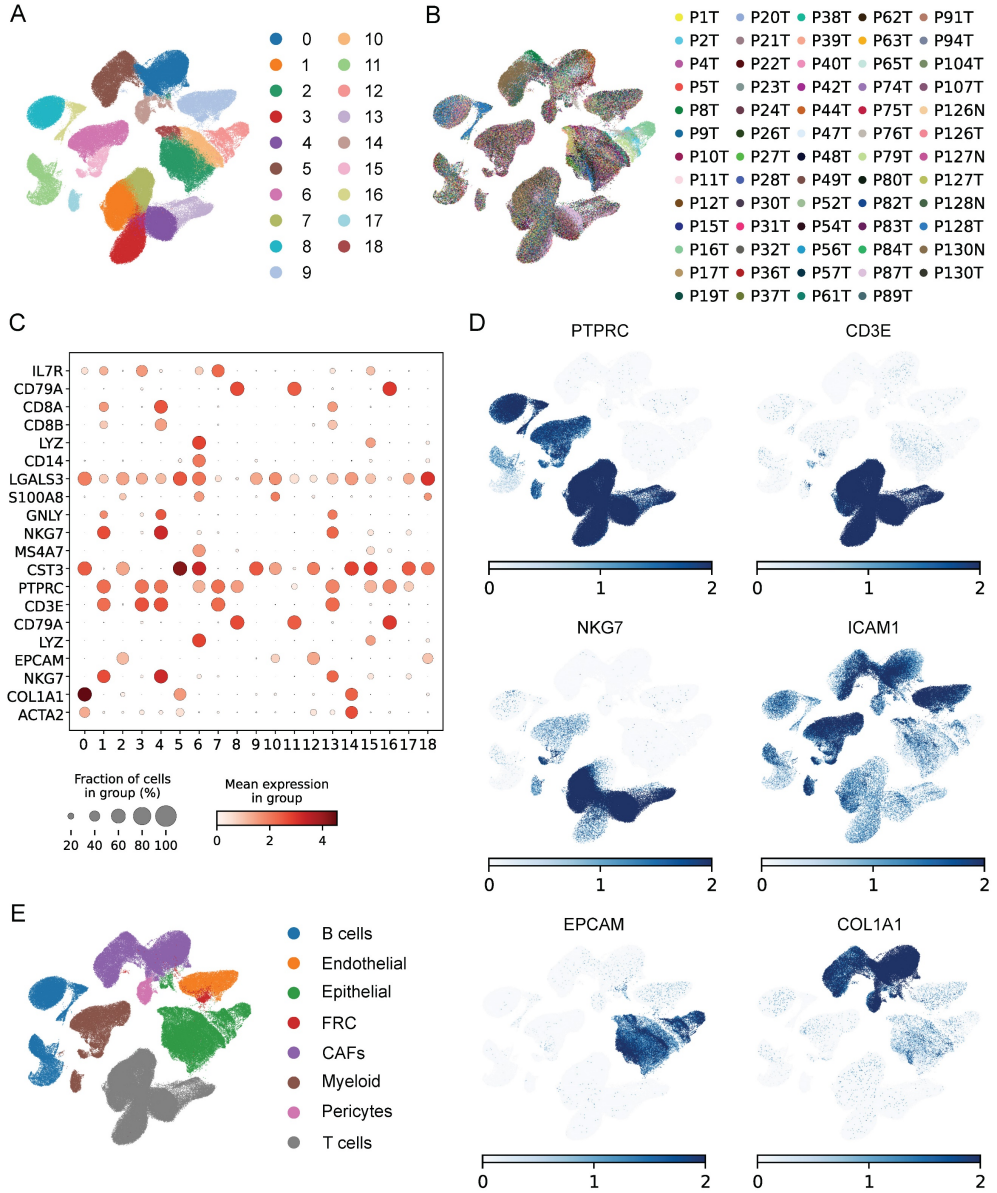
Identification of cell types by scRNA-seq analysis. (**A**) UMAP plot showing the cell clusters in GSE160269 dataset. (**B**) Excluding batch effects between patients. (**C**) Dot plot showing the expression levels of marker genes in each cluster. (**D**) UMAP plot showing marker gene expression levels among different cell clusters. (**E**) Cell annotations for these clusters.

The workflow was illustrated in **Figure 1**. After initial screening, we obtained 195,571 cells from the scRNA-seq data. We then utilized the UMAP technique for dimensionality reduction and visualization purposes, thereby obtaining 19 distinctive clusters within the ESCC context (**Figure 2A**). Since this study encompassed 64 samples, we used harmony method to remove batch effect among these samples (**Figure 2B**). Subsequently, the cells were classified based on the expression levels of the established marker genes for known cell types (**Figure 2C-D**), and 8 clusters were identified, including B cells, T cells, epithelial cells, endothelial cells, CAFs, pericytes, myeloid and fibroblastic reticular cells (FRC) (**Figure 2E**).

### Characterization of CAFs heterogeneity in terms of cellular senescence

**Figure 3:**
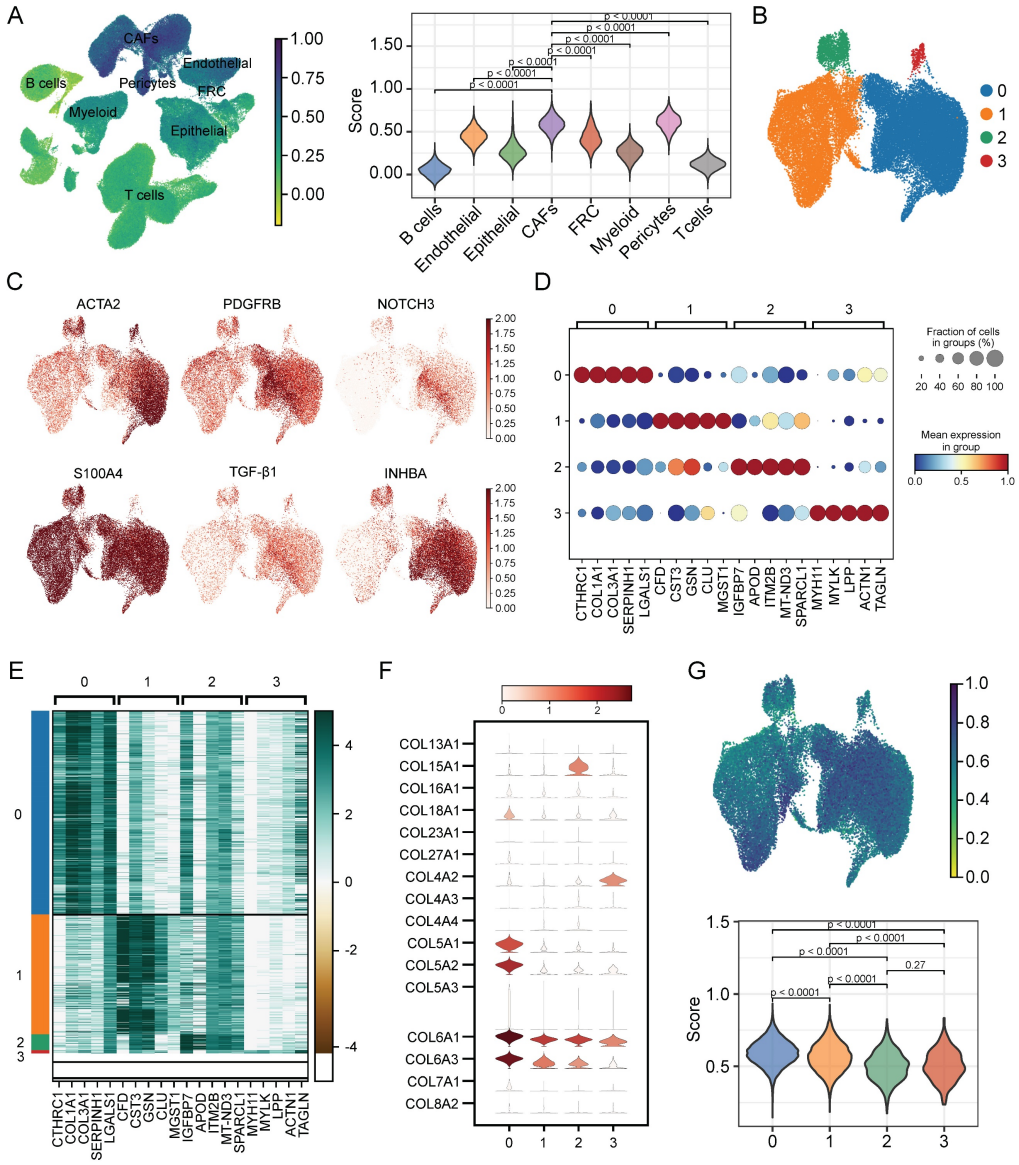
Assessing the senescence levels of individual cell types and characterization of CAFs subsets. (**A**) The expression level of FRIDMAN.SENESCENCE.UP gene set in each cluster. (**B**) UMAP plot showing distributions of four CAFs subsets. (**C**) UMAP plots showing marker gene expression levels among CAFs subsets. (**D-E**) Dot plot and heatmap showing the average expression levels of the top 5 genes in the four CAFs clusters. (**F**) Violin plots showing collagen gene expression level in the four CAFs clusters. (**G**) The expression level of FRIDMAN.SENESCENCE.UP gene set in each cluster.

To evaluate the senescence status of cell populations in ESCC, we adopted a senescence-related gene set (FRIDMAN.SENESCENCE.UP) and calculated the senescence score of each cell. Cellular senescence was distributed in a variety of cell clusters, where CAFs and pericytes showed significantly higher senescence levels than others (**Figure 3A**), and CAFs were extracted for further analysis. Then, only samples capturing more than 100 CAFs were kept to avoid the bias caused by sample heterogeneity, which reduced the sample size to 47. Further clustering analysis identified 4 CAFs subpopulations (**Figure 3B**), which had distinct marker gene expression patterns as shown in **Figure 3C**. The top 5 differentially expressed genes (DEGs) in each subpopulation were respectively displayed in dot diagram and heatmap (**Figure 3D-E**). Moreover, we found that each subtype had the individual collagen repertoire. For example, cluster 0 expressed the highest levels of *COL5A1*, *COL5A2*, *COL6A1*, *COL6A3*, and *COL18A1*, cluster 2 expressed the highest level of *COL15A1* and cluster 3 exhibited the highest level of *COL4A2* (**Figure 3F**).

**Figure 4:**
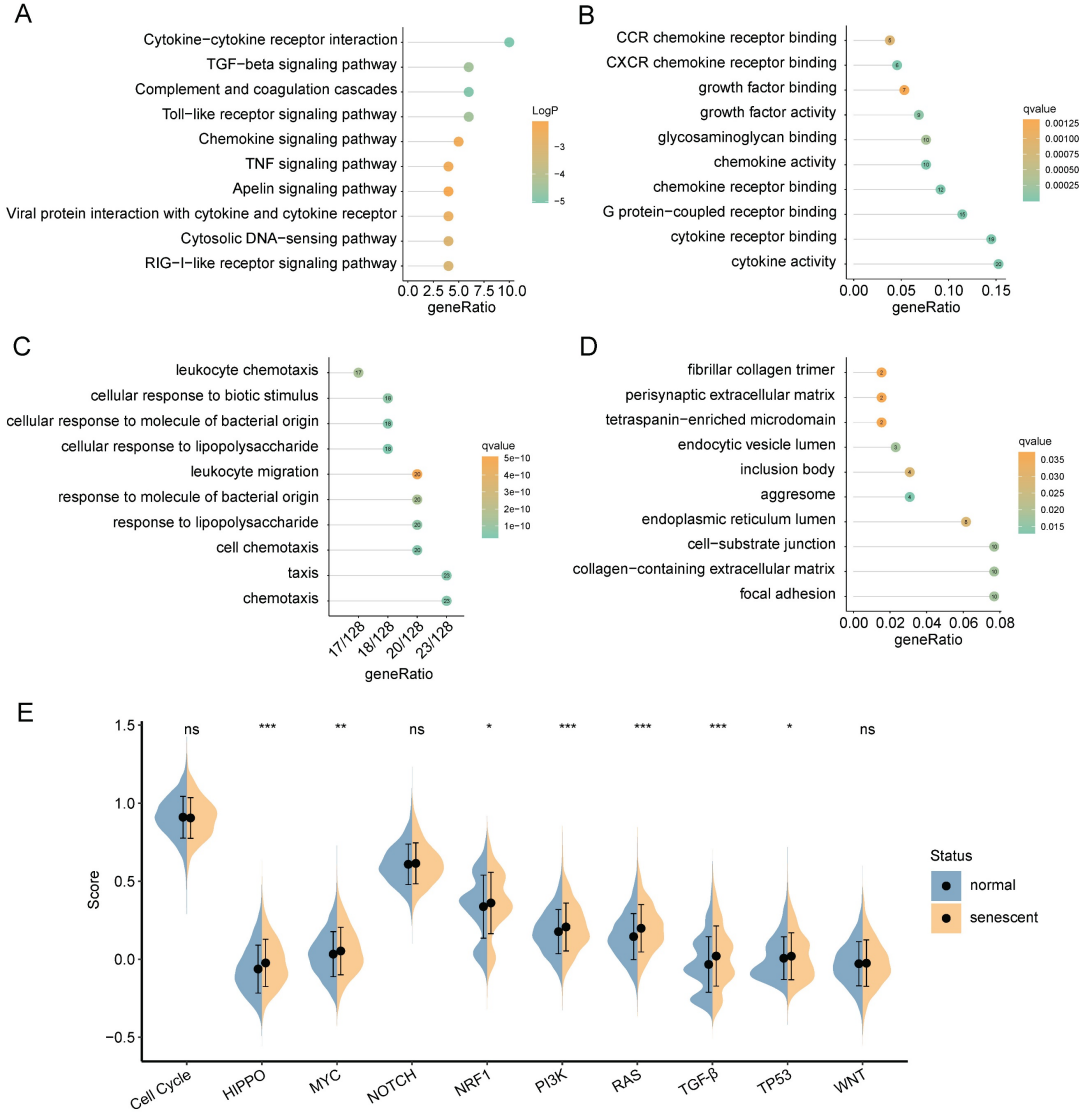
Functional enrichment analysis of DEGs between normal and senescent CAFs. (**A**) KEGG analysis. (**B-D**) GO-MF, GO-BP and GO-CC enrichment analysis. (**E**) GSEA analysis of the association between oncogenic signaling pathways and CAFs. *, *p*<0.05; **, *p*<0.01; ***, *p*<0.001; ns, no significance.

Next, we explored the degree of cellular senescence among the 4 CAFs subclusters. As shown in **Figure 3G**, cluster 0 displayed the highest senescence level, while cluster 2 exhibited the lowest average senescence score. These findings suggested the existence of heterogeneity in CAFs, which may be ascribed to the numerous origins. To explore the relationship between cellular senescence status and gene expression, we divided CAFs into senescent and normal CAFs. Subsequently, the results showed that there were totally 217 DEGs between the two groups, among which 134 were up-regulated, while other 83 were down-regulated. The KEGG pathway analysis revealed a predominant enrichment of DEGs in areas such as cytokine-cytokine receptor interaction, complement and coagulation cascades, and toll-like receptor signaling pathway (**Figure 4A**).

GO functional analysis was composed of three components: molecular functions (MF), biological processes (BP), and cellular components (CC). The top-involved MF included cytokine activity, cytokine receptor binding, and chemokine receptor binding (**Figure 4B**). The highly enriched GO terms for BP were cellular response to lipopolysaccharide, cellular response to molecule of bacterial origin, and cell chemotaxis (**Figure 4C**). Regarding CC aggresome, endocytic vesicle lumen, and focal adhesion were significantly enriched (**Figure 4D**). Subsequently, we analyzed GSEA scores of ten oncogenic signaling pathways between normal and senescent CAFs 34. The results revealed that senescent CAFs got higher scores in HIPPO, MYC, NRF1, PI3K, RAS, TGF-β, and TP53 pathways compared with normal CAFs, implying that senescent CAFs were closely related to tumor development (**Figure 4E**).

### Cell-cell interaction networks of senescent CAFs

**Figure 5:**
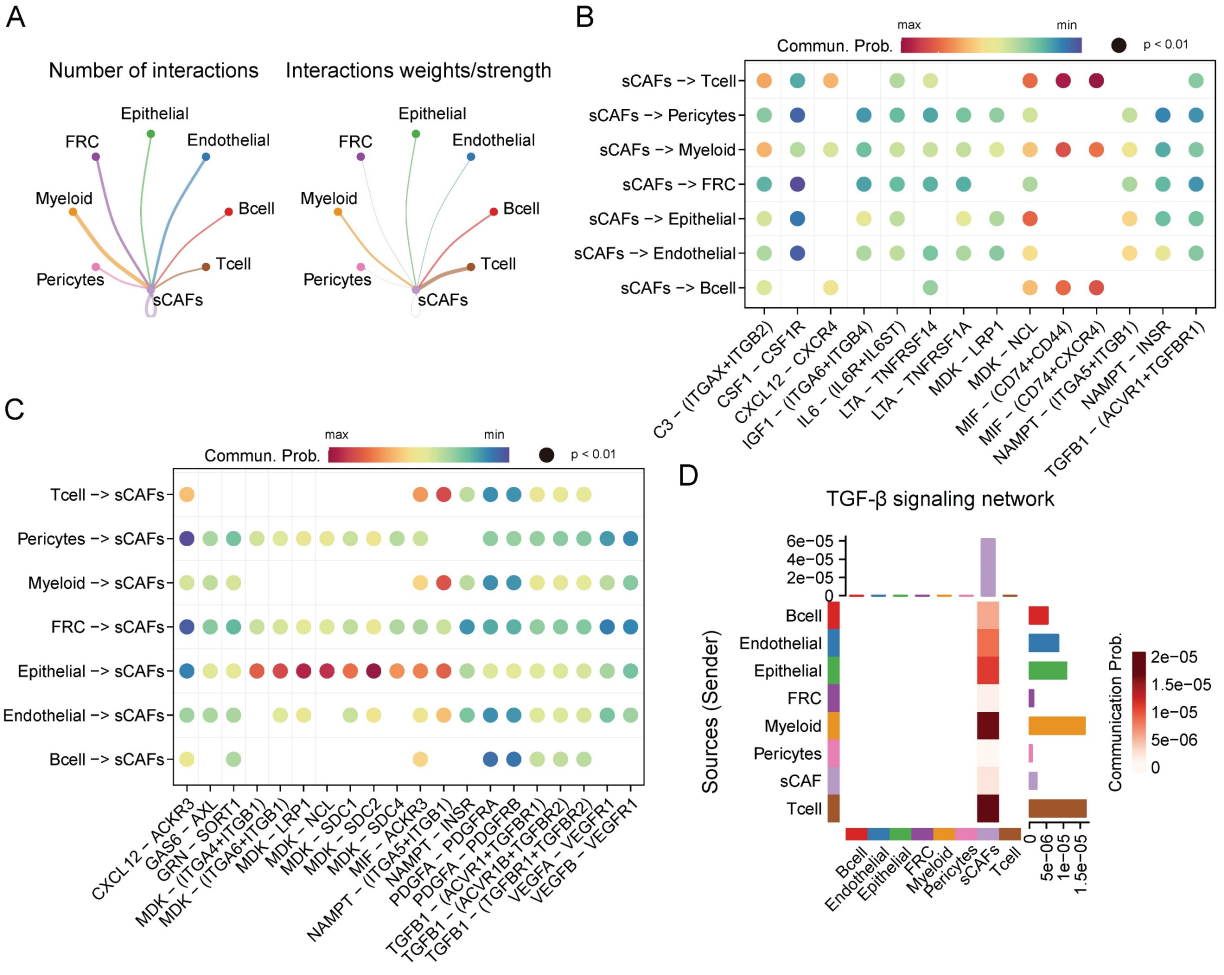
CellChat analysis among cell clusters. (**A**) The number and weights/strength of interactions between senescent CAFs and other cell types. (**B-C**) Dot plot showing ligand-receptor pairs between senescent CAFs and other cell types. (**D**) Heatmap showing communication probability of TGF-β pathway.

Given the crucial role of senescent CAFs in tumor progression, we conducted cellular communication analysis. As shown in **Figure 5A**, senescent CAFs displayed extensive communication with other cell types, especially T cells, myeloid, B cells, and epithelial cells. Next, we analyzed ligand-receptor pairs between senescent CAFs and the TME and visualized them by dot plots (**Figure 5B-C**). The results depicted that *MIF*-*CD74*/*CXCR4*/*CD44* was highly active in the interaction between senescent CAFs and immune cells. In addition, senescent CAFs established contacts with epithelial cells via *MDK*-*NCL* and *NAMPT*-(*ITGA5* + *ITGB1*). Similarly, *MDK* and its corresponding receptors (*ACKR3*, *SDC4*, *SDC2*, *SDC2*, *NCL*, *LRP1*, *ITGA6* + *ITGB1*, and *ITGA4* + *ITGB1*) activated remarkably in the signal flow from epithelial cells to senescent CAFs. Moreover, we found that senescent CAFs were the main target of TGF-β signaling pathway (**Figure 5D**), an essential node for CAFs differentiation 35.

### Construction and validation of a risk model based on CAFs senescence

**Figure 6:**
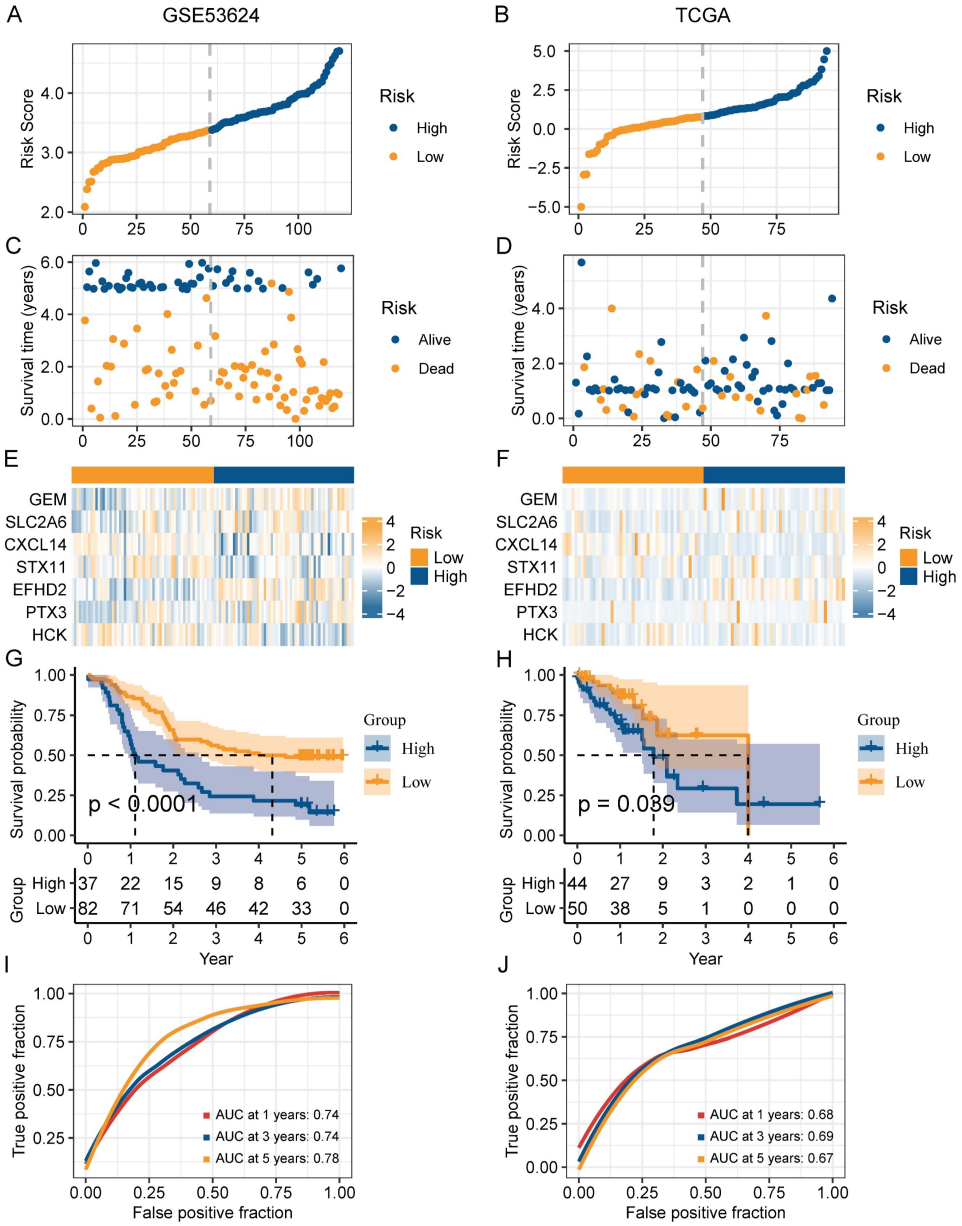
Validation of the prognostic value of CAF.SENESCENCE.SIG. (**A-B**) Risk score distribution in GSE53624 and TCGA cohorts. (**C-D**) Survival distribution in GSE53624 and TCGA cohorts. (**E-F**) Heatmap showing signature gene expression levels in high- and low-risk groups in GSE53624 and TCGA cohorts. (**G-H**) Kaplan-Meier analysis of patients in GSE53624 and TCGA cohorts. (**I-J**) ROC curves of 1-, 3-, and 5-year survival for the risk signature in GSE53624 and TCGA cohorts.

In order to narrow down the range of candidate genes, we performed the univariate Cox regression to test the 217 senescence-associated genes by evaluating the effect of each gene on the prognosis of ESCC (**Figure S1A**). To avoid omitting information that masked by complex co-expression, genes with *p* < 0.2 were subjected to further analysis. Then, we adopted LASSO and multivariate Cox analysis to screen out the 7 prominent genes (*GEM*, *SLC2A6*, *CXCL14*, *STX11*, *EFHD2*, *PTX3*, and *HCK*), and calculated the coefficient of each gene (**Figure S1B-C**). The equation for the risk model (named as CAF.SENESCENCE.SIG) was as follows: risk score = + 0.13857990 * *SLC2A6* - 0.24928075 * *GEM* - 0.07930229 * *CXCL14* - 0.34867227 * *STX11* + 0.54549264 * *EFHD2* + 0.01091528 * *PTX3* - 0.26066936 * *HCK*. Subsequently, ESCC patients in GSE53624 and TCGA datasets were divided into two groups (**Figure 6A-B**). In contrast to patients with lower risk metrics, patients with higher risk scores showed higher mortality (**Figure 6C-D**). The expression of 7 senescence-associated genes in two groups was displayed in the heatmap (**Figure 6E-F**). Moreover, Kaplan-Meier survival analysis was conducted to evaluate the predictive significance of the model. The findings indicated that in comparison to patients in the high-risk group, patients in the low-risk group had a better outcome (**Figure 6G-H**). Meanwhile, we performed ROC analysis to test the prognostic model. Both cohorts (GSE53624 and TCGA) displayed good AUC values, indicating that the signature had excellent prognostic prediction accuracy (**Figure 6I-J**).

### Clinical value evaluation of CAF.SENESCENCE.SIG

**Figure 7:**
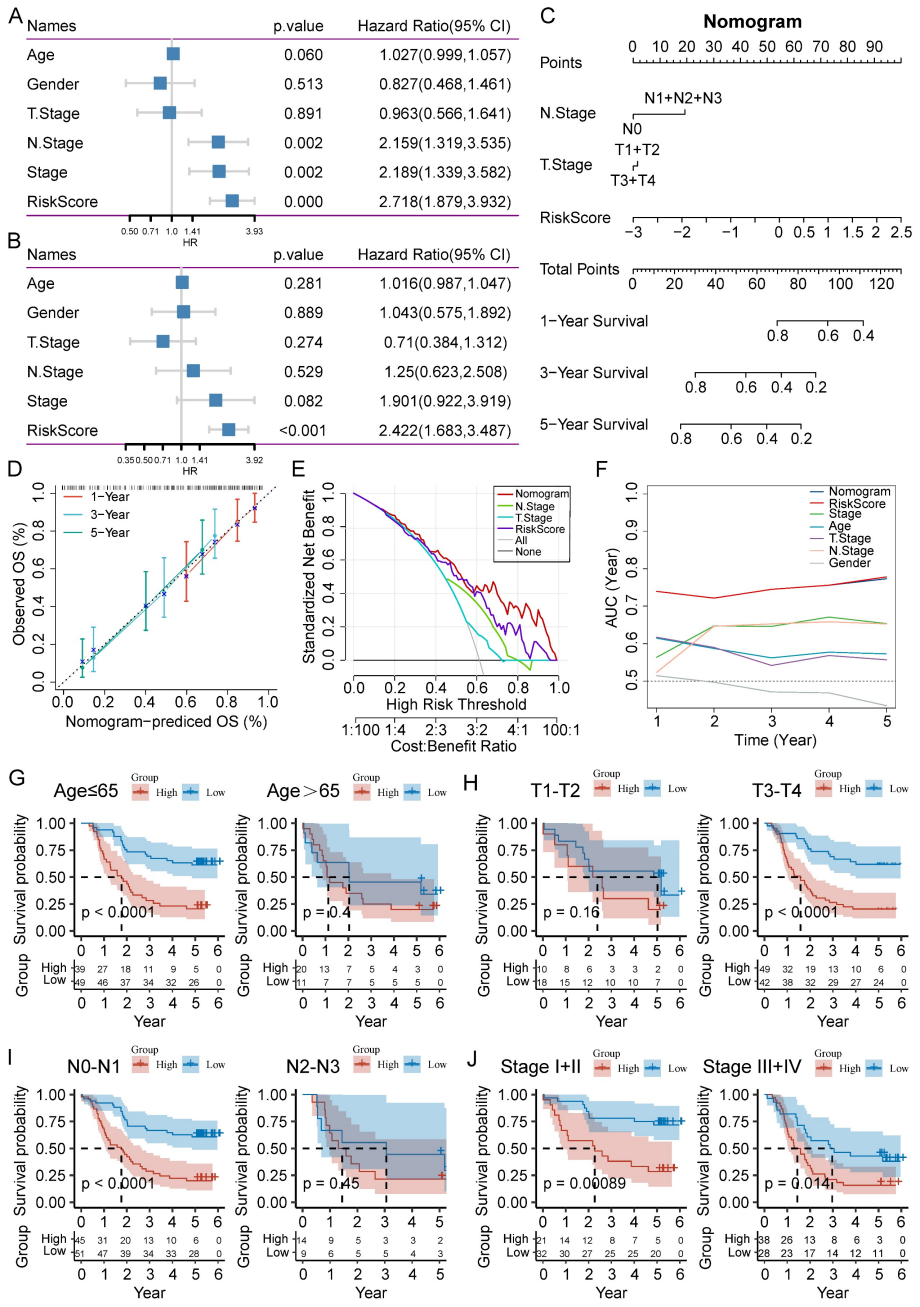
Establishment of a nomogram and survival analysis based on clinicopathological characteristics. (**A-B**) Univariate and multivariate Cox analysis of clinicopathological factors and risk score. (**C**) Establishment of the nomogram integrating N stage, T stage, and risk score. (**D**) Calibration curves for 1-, 3-, and 5-year survival of nomogram. (**E**) DCA for nomogram. (**F**) TimeROC analysis comparing the predictive capacity of the nomogram and clinicopathological factors. (**G-J**) Kaplan-Meier curves of survival differences sorted by age, T stage, N stage, and AJCC stage between risk subgroups.

To improve the performance of the signature, we incorporated the risk score and clinicopathological features through Cox regression analyses. We found that N stage, AJCC stage, and risk score could forecast OS independently (**Figure 7A**). The independent association between the risk score and OS was also observed in the multivariate Cox regression (*p*-value < 0.001), suggesting that the risk model has good reproducibility (**Figure 7B**). Then, we constructed a comprehensive nomogram that incorporated N stage, T stage, and the risk score (**Figure 7C**). The nomogram model predicted a range of 0.2 to 0.8 survival probability of patients, and the risk signature made the greatest contribution to prognosis. The calibration curve revealed that the nomogram was capable for predicting the actual survival outcomes (**Figure 7D**). In addition, DCA demonstrated that our nomogram had better predictive ability compared with N stage or T stage (**Figure 7E**). We next calculated the AUC of the nomogram, risk score, and clinicopathological features, among which the nomogram and risk score had the highest AUC value (**Figure 7F**).

To further analyze the applicability of the signature, we regrouped the ESCC patients based on different clinicopathological parameters, including age (≤65 and >65 years), T stage (T1-T2 and T3-T4), N stage (N0-N1 and N2-N3), and AJCC stage (I-II and III-IV). Survival analysis revealed that ESCC patients in the high-risk group had worse outcome than those in the low-risk group in the most cohorts, except for age (>65), T1-T2 stage, and N2-N3 stage cohorts (**Figure 7G-J**).

### Tumor immune landscape based on CAF.SENESCENCE.SIG

**Figure 8:**
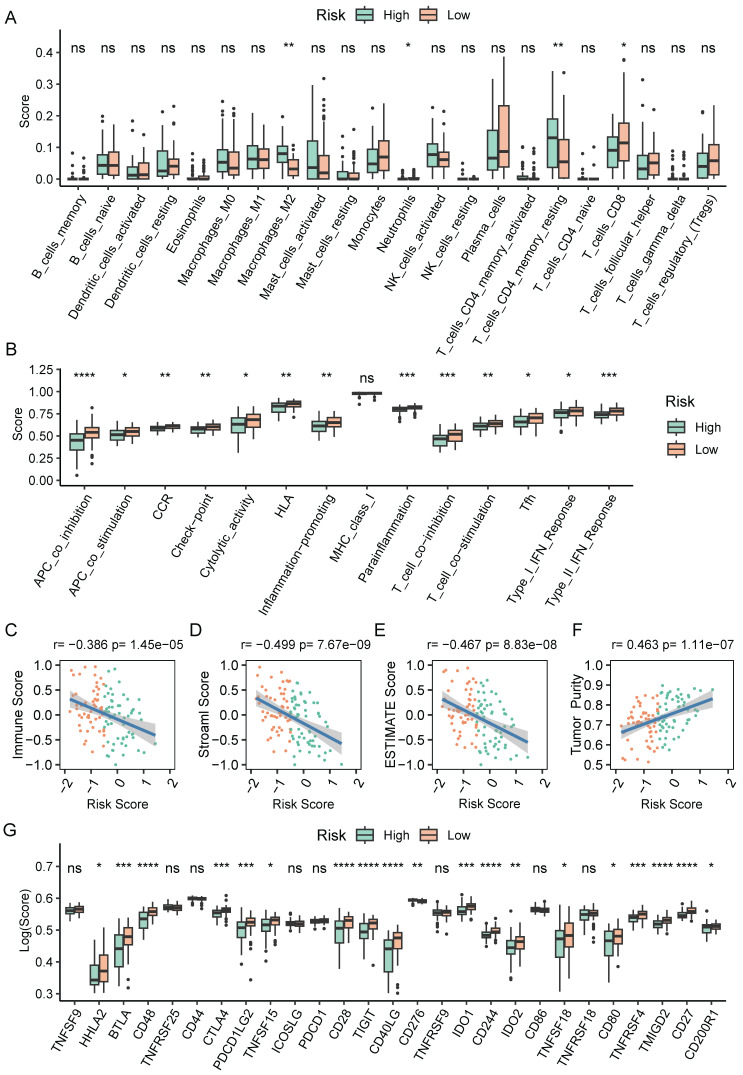
The immune cell infiltration analysis based on CAF.SENESCENCE.SIG. (**A**) Boxplot comparing the abundances of immune cells according to the risk scores. (**B**) Boxplot comparing the activities of immune-related pathways according to the risk scores. (**C-F**) Analysis of the correlation between risk scores and immune score, stromal score, ESTIMATE score, and tumor purity. (**G**) Boxplot comparing the expression levels of immune checkpoints according to the risk scores. *, *p*<0.05; **, *p*<0.01; ***, *p*<0.001; ****, *p*<0.0001; ns, no significance.

To further explore causes of survival differences, we analyzed the immune characteristics between the two subgroups. Initially, we utilized CIBERSORT to examine the enrichment scores of 22 types of immune cells. We found that low-scored patients had a higher infiltration of neutrophils and CD8^+^ T cells, while macrophages (M2) and resting memory CD4^+^ T cells showed increased accumulation in the group with a high-risk score (**Figure 8A**). Immune-related pathways analysis demonstrated that in comparison to the group with a higher score, the low-score group displayed a more active immune response (**Figure 8B**). Additionally, we adopted ESTIMATE pipeline to measure immune infiltration levels, and the results showed that the risk score was negatively associated with the immune score, stromal score, and estimate score (**Figure 8C-E**). Contrarily, **Figure 8F** showed a noteworthy positive association between the tumor purity and the risk score. Moreover, patients of high-risk group exhibited a significantly lower expression of many immune checkpoint genes (**Figure 8G**).

### Analysis of immunotherapy response based on CAF.SENESCENCE.SIG

**Figure 9:**
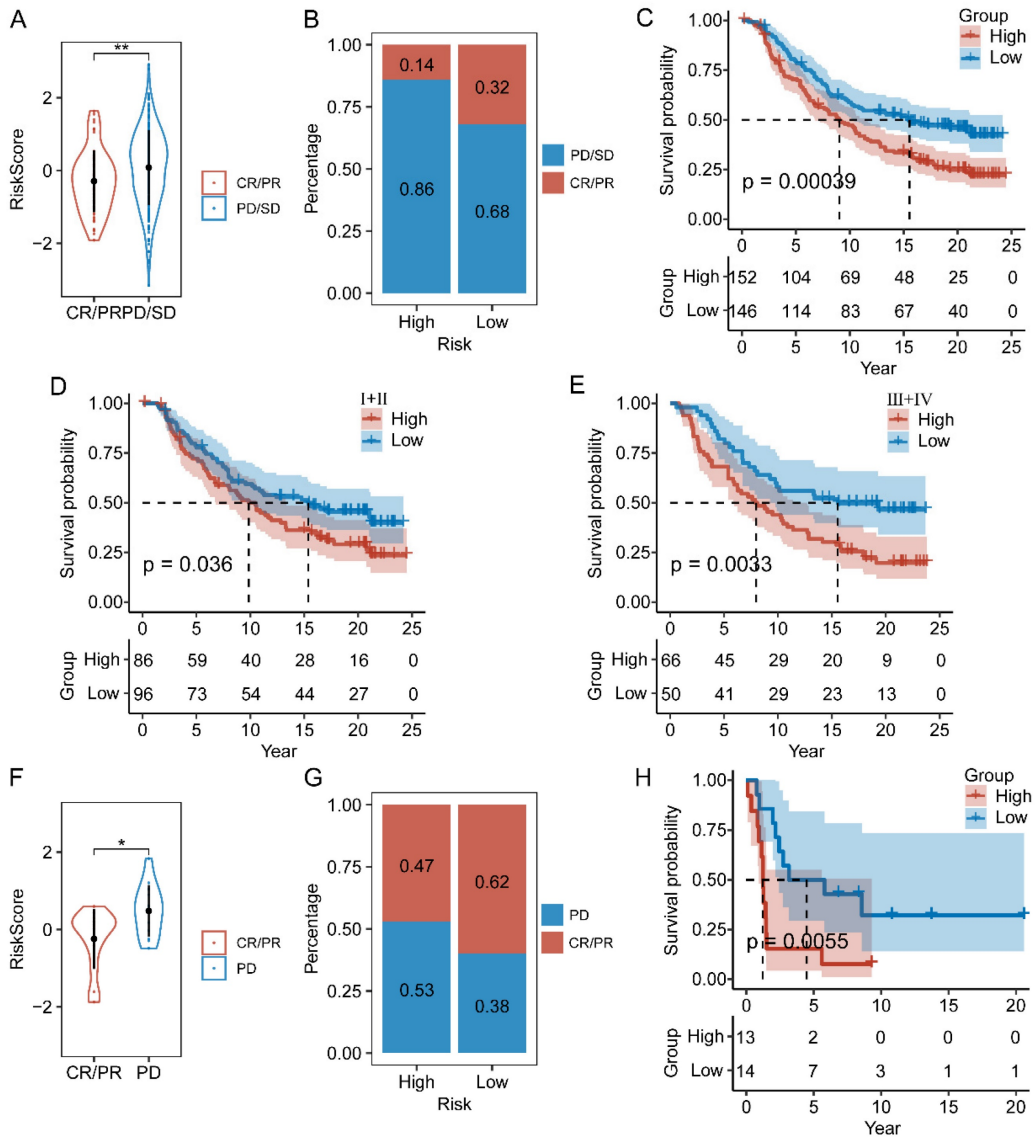
The relationship between CAF.SENESCENCE.SIG and immunotherapy response. (**A**) Differences in immunotherapy responses based on risk scores in the IMvigor210 cohort. (**B**) Differences in the distribution of immunotherapy responses in the IMvigor210 cohort. (**C**) Kaplan-Meier curves of risk subgroups in the IMvigor210 cohort. (**D**) Kaplan-Meier curves of risk subgroups in stage I-II of the IMvigor210 cohort. (**E**) Kaplan-Meier curves of risk subgroups in stage III-IV of the IMvigor210 cohort. (**F**) Differences in immunotherapy responses based on risk scores in the GSE78220 cohort. (**G**) Differences in the distribution of immunotherapy responses in the GSE78220 cohort. (**H**) Kaplan-Meier curves of risk subgroups in the GSE78220 cohort. *, *p* < 0.05; **, *p* < 0.01.

We next assessed the efficacy of the prognostic prediction in CAFs senescence-related genes for immunotherapy. In the IMvigor210 cohort, patients responded differently to anti-PD-L1 treatment, including complete response (CR), partial response (PR), stable disease (SD), and progressive disease (PD). Patients in the CR/PR response group obtained lower risk scores than those in the PD/SD group (**Figure 9A**). Meanwhile, the fraction of responders in the high-risk population fell behind that of the low-risk group (**Figure 9B**). As shown by the Kaplan-Meier curve, patients from the high-risk group had worse OS than those in the low-risk group (**Figure 9C**). Additionally, it was worth noting that stage I+II and III+IV patients showed similar survival difference between high- and low-risk groups (**Figure 9D-E**). Then, we compared the risk score between the responsive group (CR/PR) and the non-responsive group (PD) in the GSE78220 cohort, and got similar results as IMvigor210 (**Figure 9F**). Patients of low-risk group showed a positive response to anti-immune checkpoint treatments as indicated by lower incidence of disease progression (**Figure 9G**). Furthermore, the survival rate of patients with high-risk scores was considerably lower than that of patients in the low-risk group (**Figure 9H**).

### Drug sensitivity prediction

**Figure 10:**
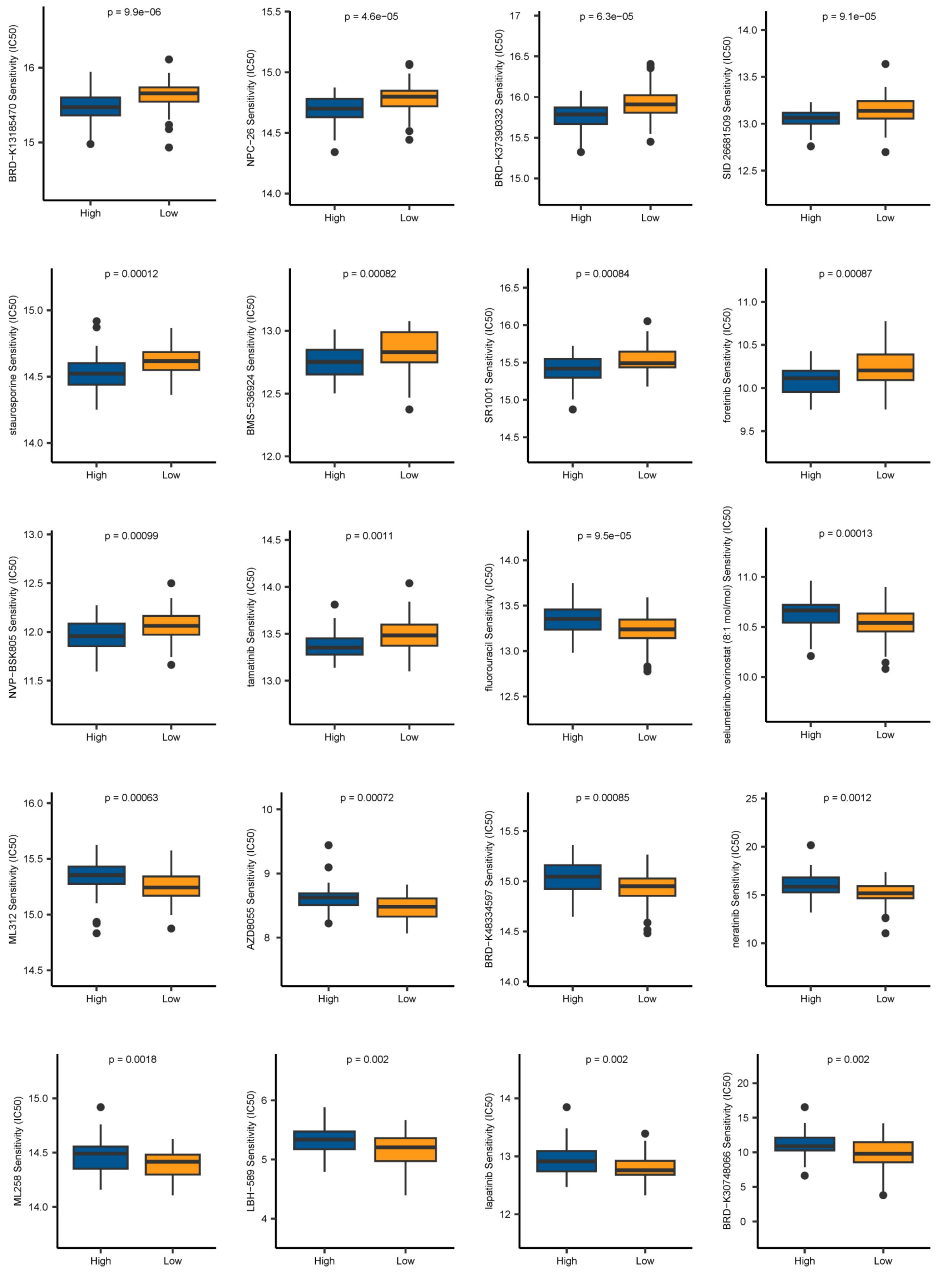
Chemotherapy drug sensitivity prediction based on CAF.SENESCENCE.SIG.

Aiming to expand the application of our signature in the field of clinical medicine, we evaluated the association between the risk scores and drug sensitivity, indicated by IC50 values. We identified 59 drugs that showed higher IC50 values in the low-risk group, and 42 drugs showing higher IC50 values in the high-risk group. The top 10 drugs ranked by *p*-values were depicted in **Figure 10**. It revealed that patients belonging to the high-risk group were more sensitive to BRD-K13185470, NPC-26, BRD-K37390332, SID 26681509, staurosporine, BMS-536924, SR1001, foretinib, NVP-BSK805, and tamatinib. Additionally, compounds such as fluorouracil, selumetinib, vorinostat, ML312, AZD8055, BRD-K48334597, neratinib, ML258, LBH-589, lapatinib, and BRD-K30748066, may be appropriate treatment for patients from the low-risk group. Together, these findings revealed that CAF.SENESCENCE.SIG could provide tailored therapeutic strategies for ESCC patients.

### *In vitro* experimental validation

**Figure 11:**
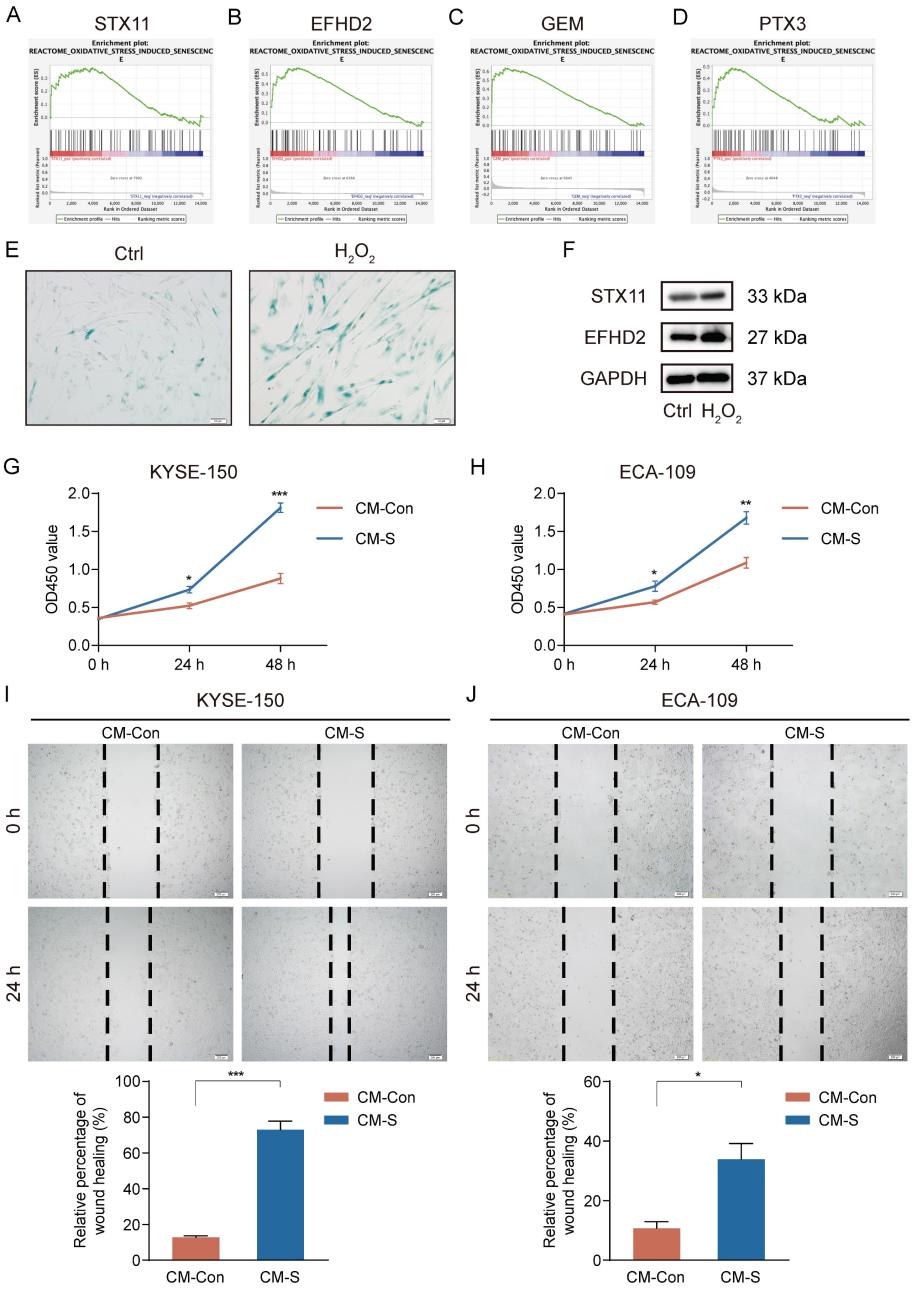
Induction of senescence in MRC-5 cells and the effect of senescent MRC-5 cells on the malignant behavior of ESCC cells *in vitro*. (**A-D**) GSEA analysis of the association between the oxidative stress-induced senescence and signature genes. (**E**) SA-β-gal staining of control and H_2_O_2_-treated MRC-5 cells. Original magnification, 200 ×. Scale bar = 50 μm. (**F**) Western blot analysis of STX11, EFHD2, and GAPDH in control and H_2_O_2_-treated MRC-5 cells. (**G-H**) CCK-8 analysis of the cell viability in ESCC cells treated with CM-Con or CM-S. (**I-J**) Wound-healing ability of ESCC cells treated with CM-Con or CM-S. Original magnification, 100 ×. Scale bar = 200 μm. *, *p*<0.05; **, *p*<0.01; ***, *p*<0.001.

High-level oxidative stress is one of the characteristics in the TME and correlates tightly with tumor occurrence and development 36. Moreover, oxidative stress has been clarified to induce senescence in CAFs 37. Thus, we conducted GSEA to assess the connection between 7 signature genes and oxidative stress-induced senescence. Our analysis revealed a significantly positive association between *STX11*, *EFHD2*, *GEM*, and *PTX3* and oxidative stress-induced senescence (**Figure 11A-D**). We then treated MRC-5 cells with H_2_O_2_ to construct a cellular senescence model. As shown in **Figure 11E**, H_2_O_2_ treatment induced more expression of senescence-associated β-galactosidase. Additionally, the mRNA and protein levels of STX11 and EFHD2 were significantly increased after H_2_O_2_ treatment (**Figure 11F and Figure S2**). Furthermore, we collected the CM of control and senescent MRC-5 cells (named CM-Con and CM-S) to culture cancer cells. CCK-8 assay revealed that CM-S remarkably promoted ESCC cell proliferation (**Figure 11G-H**). The wound-healing assay demonstrated that the migration capacity of ESCC cells was enhanced by CM from senescent MRC-5 cells (**Figure 11I-J**). These results aligned with our bioinformatic findings, suggesting that senescent CAFs were key players in mediating tumor progression.

## Discussion

Cellular senescence, a hallmark of cancer, is widely recognized as an indispensable mediator in tumorigenesis and progression 38. In particular, senescent stromal cells in the TME have been confirmed to exhibit a secretory phenotype, thereby reinforcing the malignant phenotype of neighboring tumor cells 39. Nonetheless, the potential of clinical application and practical significance of stromal cellular senescence in ESCC are still not well understood.

In this study, we utilized the scRNA-seq data of ESCC acquired from GEO to evaluate the cellular senescence level in individual cell populations. Our results revealed that pericytes and CAFs exhibited the highest senescence level. It should be emphasized that pericytes share some cell markers with CAFs, making it difficult to clearly distinguish them at the single-cell level 40, 41. Additionally, considering the limited number of pericytes, we mainly focused on CAFs. A lot of literature has revealed that CAFs display substantial heterogeneity based on their phenotype and function 42-44. Here, we identified four CAFs subclusters expressing different markers. Moreover, the degree of their cellular senescence was also distinctly different from each other, among which cluster 0 had the highest degree of senescence. To further explore the differences in the transcriptome of senescent CAFs, we divided all CAFs into two subgroups: normal CAFs and senescent CAFs. Subsequently, 217 DEGs were identified by comparing gene expression between the two subgroups. The results revealed that senescence-related DEGs were largely enriched in tumor-associated pathways, indicating that these genes could accelerate the development of ESCC. Similarly, numerous studies have also reported the tumor-promoting effect of senescent CAFs 37, 45, 46. Hence, developing senescent CAF-associated predictive biomarkers in ESCC is of great clinical and practical significance.

Through LASSO-Cox regression analyses, a novel CAFs senescence-related risk model with 7 genes was finally established, comprising three risk associated genes (*SLC2A6*, *EFHD2*, and *PTX3*) and four protective genes (*GEM*, *CXCL14*, *STX11*, and *HCK*). Although some of these signature genes have been studied for their important functions in tumors, including ESCC, others remain to be uncovered. For example, EFHD2 promotes lung cancer cell resistance to chemotherapy through the NOX4-ROS-ABCC1 signaling, and high level of *EFHD2* is correlated with a worse survival in lung cancer patients received chemotherapy 47. *PTX3* was reported to have the potential for predicting prognosis as well as immunotherapy response in lung cancer 48. Furthermore, PTX3 can facilitate ESCC cell proliferation and migration 49. Grounded on the prognostically relevant signature, we calculated a senescence-based risk score respectively for each patient. After that, patients were classified into two subgroups, to explore the characteristics in terms of prognosis, immune landscape, immunotherapy response, and chemotherapy sensitivity. These results might provide new guidance for predicting prognosis risk and selecting treatment strategies for ESCC patients.

Immunotherapy, especially immune checkpoint inhibitors, has greatly changed the treatment pattern of ESCC 50. However, the clinical benefit of immunotherapy is still very limited 51. The clinical efficacy of immunotherapy is largely affected by both the tumor and the TME, and shows high heterogeneity among individuals 52. The TME is composed of various immune cells, such as regulatory T cells, cytotoxic T cells, and macrophages 16. CAFs, as a predominant cell type in the TME, interact extensively with these immune cells and play multifaceted roles in immune responses 53, 54. On the one hand, studies have reported that CAFs promote an immunosuppressive tumor environment mainly through the following mechanisms: (i) impairing T cells proliferation and activation, and modulating immune checkpoints expression on T cells; (ii) recruiting monocytes into the tumor and inducing their differentiation into M2-like polarized macrophages; and (iii) secreting ECM components, thus forming a tough barrier against anti-tumor immune cells infiltration 54-56. On the other hand, accumulating evidence indicates the presence of immunostimulatory CAFs, which enhance anti-tumor immune responses through preventing T cell exhaustion and promoting plasma cell dissemination 57, 58. However, the role of senescent CAFs in immune microenvironment remodeling remains largely unknown. In our study, we characterized the immune landscape in different groups, and found that the patients with high-risk scores were in relevance with low immune infiltration, low expression of immune checkpoints, and high tumor purity, corresponding to an immune-desert phenotype 59. Moreover, CD8^+^ T cells, which mediate anti-tumor responses in the immune system, displayed decreased infiltration levels in patients with high-risk scores, accompanied by elevated levels of M2 macrophages infiltration. Combining above results, we inferred that immunotherapy was more likely to benefit patients of the low-risk group, and this inference was fully verified in the immunotherapy cohorts.

Although we primarily focused on cellular senescence of CAFs, other cells including endothelial cells, macrophages, and T cells also undergo senescence-related alterations, thereby affecting tumor progression 60. Senescence in endothelial cells is identified in many types of solid tumors, and is significantly correlated with tumor proliferation and dissemination 61, 62. Furthermore, previous studies have revealed that senescent macrophages promote lung cancer progression at early stages through secreting SASP factors and suppressing cytotoxic T cell responses 63. Researchers also demonstrate that removing senescent macrophages remarkably improves survival outlooks in mouse models of lung cancer 64. In addition, senescent T cells fail to effectively recognize and eliminate tumor cells, leading to a suppressive microenvironment 65. Consequently, cellular senescence could be applied as prognostic markers and promising therapeutic targets.

Importantly, there are several limitations in this study. Firstly, due to the lack of open data on ESCC patients receiving immunotherapy, we could only analyze the immunotherapy response in other cancer cohorts. Secondly, because of the difficulty of isolation and culture of primary CAFs, we referred to a previous study using MRC-5 cells to perform *in vitro* studies 66. Thirdly, despite of the large scale of data, it's necessary to remind that our study mainly relied on transcriptome level information, which may limit the practical application of our signature for peptides test field. Therefore, the prognostic significance of the signature genes still needs to be verified at histological level, and subsequent studies are needed to illustrate their biological functions and relevant mechanisms.

## Supplementary Material

Supplementary figures.

## Abbreviations

CAFs: cancer-associated fibroblasts
ESCC: esophageal squamous cell carcinoma
EC: esophageal carcinoma
TME: tumor microenvironment
SASP: the senescence-associated secretory phenotype
scRNA-seq: single-cell RNA sequencing
RNA-seq: RNA sequencing
GO: gene ontology
KEGG: kyoto encyclopedia of genes and genomes
GSEA: gene set enrichment analysis
LASSO: least absolute shrinkage and selection operator
OS: overall survival
ROC: receiver operating characteristic
AUC: area under the ROC curve
DCA: decision curve analysis
FRC: fibroblastic reticular cells
DEGs: differentially expressed genes
MF: molecular functions
BP: biological processes
CC: cellular components
CR: complete response
PR: partial response
SD: stable disease
PD: progressive disease
IC50: half maximal inhibitory concentration
SA-β-gal: senescence associated β-galactosidase
qRT-PCR: quantitative real-time polymerase chain reaction
CM: conditioned medium
CCK-8: cell counting kit-8
