# Supplementary material for: Integrative Analysis and Validation of a Cancer-associated Fibroblasts Senescence-related Signature for Risk Stratification and Therapeutic Prediction in Esophageal Squamous Cell Carcinoma
Source: J Cancer. 2024 Sep 9;15(17):5742–61. doi: 10.7150/jca.100430 (PMC11414623; doi:10.7150/jca.100430)
Supplement: Supplementary file 1 — Supplementary figures. [file jcav15p5742s1.pdf]

Supplementary figure

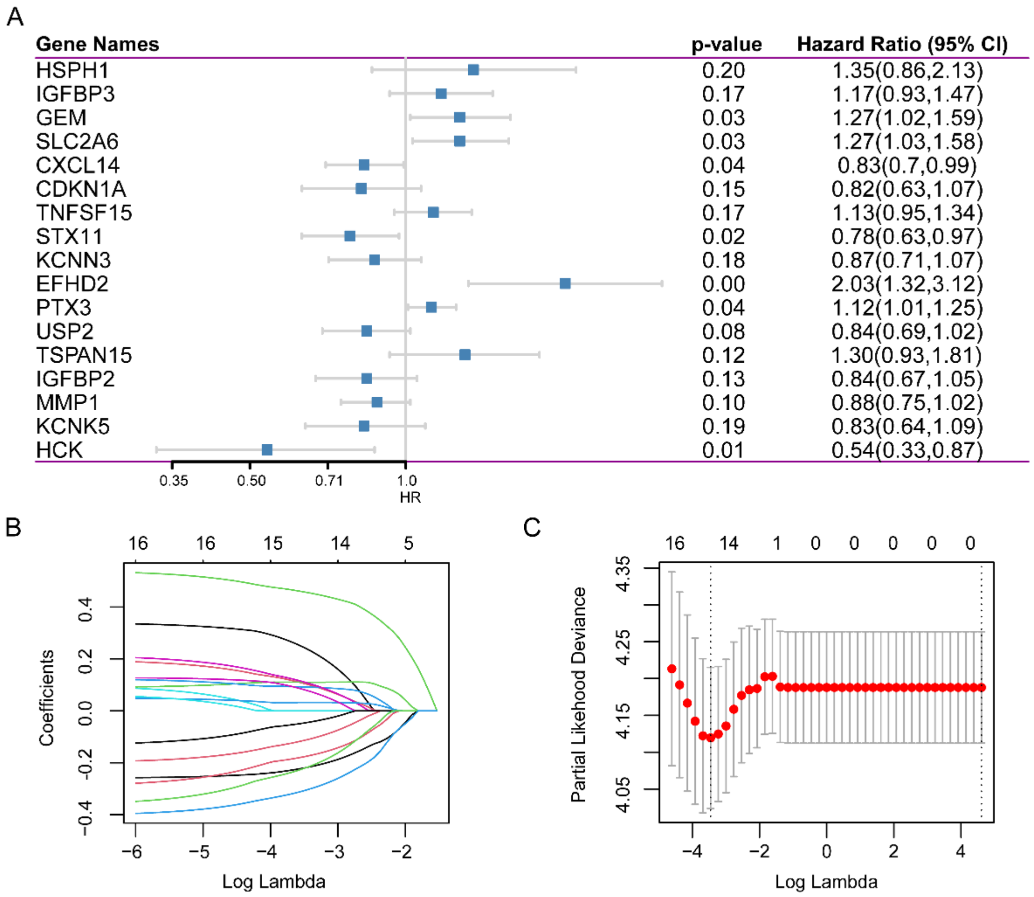

Figure S1. Identification of prognostic genes. (**A**) The univariate Cox analysis screened 17 prognostic CAFs senescence-related genes. (**B**) The LASSO regression analysis. (**C**) The partial likehood deviance of the signature.

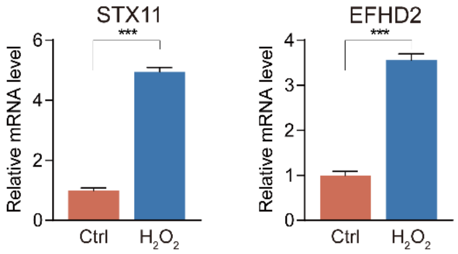

Figure S2. qRT-PCR analysis of the expression of *STX11* and *EFHD2* in control and H_2O_2-treated MRC-5 cells. ***, $p < 0.001$.
